# Responses of Periphyton Microbial Growth, Activity, and Pollutant Removal Efficiency to Cu Exposure

**DOI:** 10.3390/ijerph17030941

**Published:** 2020-02-03

**Authors:** Wei Zhong, Weiqun Zhao, Jianhui Song

**Affiliations:** 1 College of Environment, Hohai University, 1 Xikang Road, Nanjing 210098, China; 2 Power China Kuminng Engineering Co., Ltd., Kuminng 650051, China; zhaoweiqun_kmy@powerchina.cn; 3 Sinohydro Bureau 8 Co., Ltd., Changsha 410004, China; song523168@163.com

**Keywords:** periphyton, metal pollution, Cu^2+^, bioremediation, carbon sources utilization

## Abstract

Periphyton is an effective matrix for the removal of pollutants in wastewater and has been considered a promising method of bioremediation. However, it still needs to be verified whether periphyton can maintain microbial activity and pollutant removal efficiency when dealing with the influence with complex components, and the underlying mechanisms of periphyton need to be revealed further. Herein, this study investigated the microbial growth, activity and functional responses of periphyton after removal of Cu from wastewater. Results showed that the cultivated periphyton was dominated by filamentous algae, and high Cu removal efficiencies by periphyton were obtained after 108 h treatments. Although 2 mg/L Cu^2+^ changed the microalgal growth (decreasing the contents of total chlorophyll-a (Chla), the carbon source utilization and microbial metabolic activity in periphyton were not significantly affected and even increased by 2 mg/L Cu^2+^. Moreover, chemical oxygen demand (COD) removal rates were sustained after 0.5 and 2 mg/L Cu^2+^ treatments. Our work showed that periphyton had strong tolerance and resistance on Cu stress and is environmentally friendly in dealing with wastewater containing heavy metals, as the microbial functions in pollutant removal could be maintained.

## 1. Introduction

Bioremediation is an important way to control or remove the amount of hazardous waste at pollutant places using the aggregations of microorganisms [1,2]. Bioremediation is generally conducted in non-sterile open environments which have different bacteria [3,4]. Previous studies have divided bioremediation methods into two ways based on the degradation places, ex situ and in situ bioremediation, and the later one is primarily addressed [2]. Having been used and studied for several decades, however, the in situ bioremediation technology has not received the results as expected and was not very successful due to the limitations of the decreased ecological steadiness under changing environments [1,5]. Hence, there is still a need to exploit a steady microbial ecosystem which can maintain the microbial structure and function during bioremediation work being carried out.

Among the various biological methods, periphyton filtration is an environmentally friendly technology and a promising method of bioremediation [6,7]. Periphyton is ubiquitous in aquatic environments mainly consisting of algae, fungi and bacteria [8,9]. Periphyton has been shown to be capable of improving the water quality by adsorpting and trapping heavy metals and organic molecules in water [9,10,11,12]. In addition, periphyton is important for the ecological functions of aquatic environments, playing crucial roles in primary productivity, trophic transfer of nutrients and food source biomasses in aquatic ecosystems [8,13,14]. Due to these characteristics and immobilization properties, periphyton becomes an underlying regulator of extra waste materials in water environments [7,15].

Recently, periphytic biofilms have become a common biological treatment form in water and/or wastewater disposal, and work efficiently in dealing with nutrient removal and several containments such as organic matters, nanoparticles and heavy metals [16,17,18,19]. Compared with single-species communities for biological treatment, multi-species periphyton have stronger pollution load capacity and anti-interference ability [9,20,21,22]. Besides, the efficiency of commonly accepted single-species communities for pollution treatment could be easily influenced by the complex wastewater components, such as organic pollutants and heavy metals [9]. Periphyton has been demonstrated to exhibit more resistance to environmental disturbances through self-controlling the community structure and microbial activity [14,19]. However, it still needs to be verified whether periphyton can maintain the normal removal efficiency for sewage and wastewater when dealing with the influence of complex components, such as high heavy metal content water. Moreover, the underlying response mechanisms of periphyton need to be revealed in order to better use periphyton in bioremediation.

Accordingly, a study was conducted to explore the functional sustainability of periphyton after exposure to different concentrations of heavy metal. Cu^2+^ was selected as it is one of the most detected heavy metals in aquatic environments [23,24]. Periphyton was cultivated in a laboratory for 28 days and two experiments were conducted: (1) adsorption and removal of Cu^2+^ by periphyton and the responses of microbial growth activity; and (2) pollutant removal efficiency of periphyton after Cu exposure. The main objectives were: (1) to study the removal of Cu^2+^ with different concentrations by periphyton; (2) to explore the influences of Cu^2+^ on species composition (algae) and ecosystem functional capacities (carbon metabolism) in periphyton; and (3) to examine whether the organic matter removal rates of periphyton can be maintained after exposure to Cu^2+^.

## 2. Materials and Methods

### 2.1. Periphyton Culture

For periphyton culturation, acrylic carriers were used as the solid substrates in this study. Briefly, pieces of acrylic carriers (3 × 3 cm) were glued to the bottom of a tank (50 × 50 × 35 cm), which was filled with water taken from Xuanwu Lake, Nanjing. To ensure the microbial growth of periphyton, ten milliliter Woods Hole culture medium (WC medium) was introduced to 1 L lake water [25] and mixed. Thereafter, the tank was placed into a greenhouse (20 ± 1 °C, light:dark = 12:12 h). The culture experiment was conducted for four weeks, and the dynamic changes of dry weight of periphyton were monitored. Afterwards, mature and stabilized biofilms (3–4 mm thickness) were obtained and used in the following experiments.

### 2.2. Experimental Design

After periphyton cultivation, thirty pieces of acrylic carriers were carefully collected and transferred into microcosms (cylindrical plexiglass), each filled with 3 L of water (approximately 10 cm in depth) in a tank. The microcosms were placed in an indoor laboratory where a stabilized environment was secured (20 ± 0.5 °C, light:dark = 12:12 h). All the biofilm carriers were vertically placed in the microcosms.

In the experiments of Cu^2+^ removal by the periphyton, two concentrations (0.5 and 2 mg/L) of Cu^2+^ was introduced into the microcosms. CuSO_4_·5H_2_O was used to prepared a Cu stock of 500 mg/L. Afterwards, 3 and 12 mL of the stock suspension were introduced to the microcosms to obtain the final concentrations. A control test was set up without addition of Cu. At the same time, an air bubble-sample mixture was used and pumped into the microcosms. Under these conditions, water samples in microcosms were collected every 12 h for 108 h to determine the Cu^2+^ content. At the end of the exposure experiments, microalgae community structure and carbon source utilization in periphyton were also measured to study the impacts of Cu^2+^ on the microbial growth and metabolic activity of periphyton. 

After the 108 h Cu^2+^ exposure incubation, the following experiment was performed to examine the organic matter removal rates of periphyton. The periphyton were collected, transferred into the new microcosms and cultivated in synthetic wastewater for 144 h. During this period, water samples in microcosms were collected and the chemical oxygen demand was measured to represent the organic matter removal rate of periphyton after being treated with Cu. Briefly, approximately 0.3 g periphyton (wet weight) from the three Cu treatments (control, 0.5 mg/L, and 2 mg/L) were transferred to an Erlenmeyer flask filled with 150 mL of synthetic wastewater. The components of synthetic wastewater were prepared following a previous study [26]: COD 142 ± 4.5 mg/L, total nitrogen 3.74 ± 0.32 mg/L, total phosphorus 1.16 ± 0.16 mg/L. These experiments were conducted in a small tank under the same conditions as the periphyton culture. The water samples were collected at 0, 24, 48, 72, 96, 120 and 144 h to measure the concentration of COD.

### 2.3. Samples Analysis

During the culture of periphyton, the dry weight of periphyton was measured at different times and the values were expressed as g/m^2^. The morphology of periphyton was imaged by scanning electron microscope (SEM, S-4800, Hitachi, Japan) after alcohol dehydration and gold sputtering. 

In the Cu^2+^ treated experiments, water samples were collected at the different interval times, and Cu^2+^ concentration was determined using inductively coupled plasma mass spectrometry (ICP-MS) after filtration through a 0.45 µm filter. The content of chlorophyll-a (Chla) and microbial metabolic activity of periphyton were measured.

As for the Chla, one piece of carrier was removed from each tank at a different time interval (0, 24, 48, 72, 96, 120 and 144 h). The periphyton samples were scraped off using a sterile brush. Then, 0.5 g of wet periphytic biofilm was mixed in 60 mL 0.85% NaCl solution and shaken for 30 min at 150 rpm. The Chla concentration and quantum yield of periphyton suspension were determined using the PHYTO-PAM-II [27,28]. The details are provided in the Supplementary File.

The microbial metabolic activity of periphytic biofilms were measured using BiologTM ECO Microplates (Biolog Inc., Hayward, CA, USA) at 0, 12, 24, 36, 48, 60, 72, 84, 96, 108 and 120 h during the incubation [29,30]. Zero point one grams of wet biofilm sample was mixed in 50 mL of 0.85% NaCl solution and shaken for 30 min at 150 rpm. After a 10-min settling, a 150 µL aliquot was pipetted into each well of the ECO microplate and the absorbance was measured with a Synergy H4 Microplate Reader (Bio-Tek Instruments Inc., Winooski, USA) at 590 nm wavelength. The details are provided in the Supplementary File.

In this study, all the performed experiments were repeated three times, and data are shown as the means ± SD. ANOVA was used to test for differences at the significance level *p* < 0.05. As for the analysis of microbial metabolic activity, values obtained from ECO microplate lower than the control well were set to the same value as that of the control. For each condition, average well color development (AWCD) and Shannon index were calculated based on absorbance data to analyze the metabolic activity and functional richness of periphyton under different treatments.

## 3. Results and Discussion

### 3.1. Periphyton Characterizaiton and Cu^2+^ Removal Efficiency

**Figure 1:**
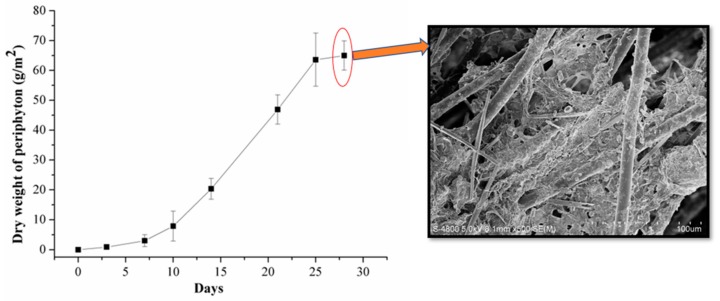
The dry weight of periphyton during the culture and the SEM image of periphyton at day 28.

During the periphyton culture, the dry weight of periphyton was measured at day 0, 3, 7, 10, 14, 21, 25 and 28. As shown in Figure 1, the microbial biomasses of periphyton were very low within the first week, and then a significant increase was observed, suggesting that periphyton began a logarithmic phase of growth. After 28 days of culture, the dry weight of periphyton reached a peak of 64.98 ± 4.89 g/m^2^. SEM was performed to observe the morphological features of periphyton at day 28. The periphyton was dominated by algal groups, especially filamentous algae, and the microbial cells were surrounded by extracellular polymeric substances.

**Figure 2:**
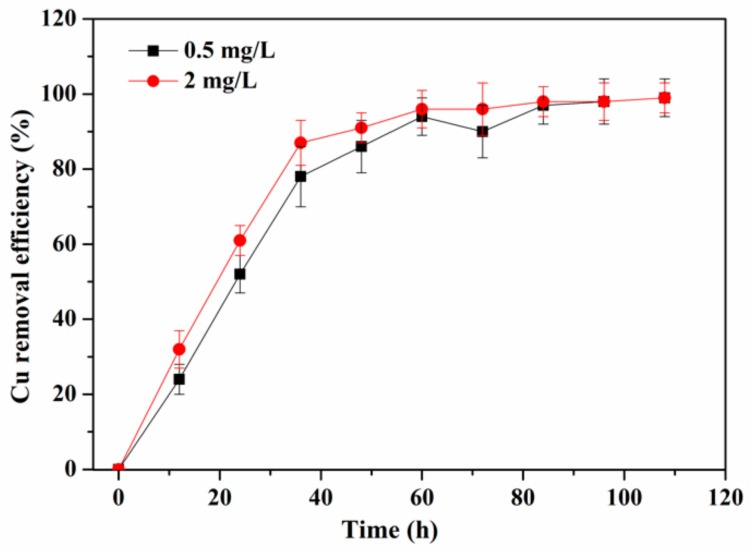
Cu^2+^ removal efficiency by the periphyton at Cu^2+^ concentrations of 0.5 and 2 mg/L.

The Cu^2+^ removal efficiency by periphyton is shown in Figure 2. Results showed that the removal of Cu^2+^ consistently increased in the first 60 h in the 0.5 and 2 mg/L Cu^2+^ exposure groups. In detail, the Cu^2+^ removal efficiency dramatically increased within the first 48 h to 86 ± 7.0% and 91 ± 4% in the 0.5 and 2 mg/L Cu^2+^ exposure groups, respectively. Then, in 48–60 h, the removal efficiency of Cu^2+^ still increased to 94 ± 5% and 96 ± 5% in the 0.5 and 2 mg/L Cu^2+^ groups, respectively, but the increased rate was obviously lower than the first 48 h. After that, Cu^2+^ removal efficiency kept relatively stable. At the end of the 108 h exposure experiment, the final Cu^2+^ removal efficiency was up to 99 ± 5% and 98 ± 4% in the 0.5 and 2 mg/L Cu^2+^ groups, respectively. The results indicated that periphyton could be effective in removing Cu^2+^ in polluted environments. Similar results were obtained by Ma et al. [25], in which the higher removal efficiency of Cu^2+^ could be attained at 98.2% using a tubular periphyton bioreactor. Accordingly, periphyton remove heavy metals by three main mechanisms: adsorption in extracellular polymeric substances, cell surface adsorption and intracellular uptake [9]. Previous studies have indicated that biosorption could be the primary mechanism of Cu^2+^ removal by periphyton and the fast removal efficiency was due to the external mass transfer, followed by intra-particle diffusion [10].

### 3.2. Effect of Cu^2+^ on Microbial Growth of Periphyton

**Figure 3:**
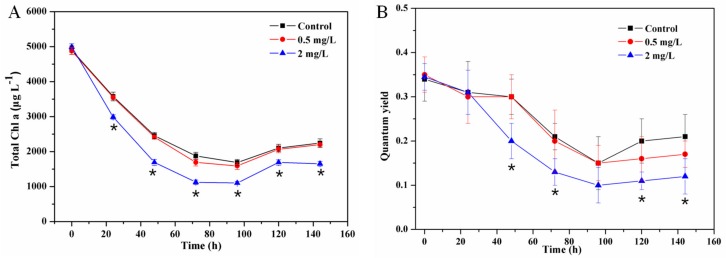
Dynamic changes of chlorophyll-a (Chla) (**A**) and quantum yield (**B**) in periphyton in different Cu treatments (0, 0.5 and 2 mg/L). * indicated significant differences at *p* < 0.05 between the control and Cu^2+^ treatments.

The microalgal biomasses (measured by Chla concentration) were determined at different interval times to evaluate the effects of Cu^2+^ on the microalgal growth in periphyton (Figure 3). The results indicated that in the first 96 h, the total Chla contents obviously decreased in the control and Cu^2+^ treated groups. Specifically, the concentrations of the total Chla decreased from 4896 ± 123 to 1687 ± 88 μg/L, from 4875 ± 98 to 1589 ± 101 μg/L, and from 4992 ± 87 to 1100 ± 45 μg/L in the control, 0.5 mg/L and 2 mg/L Cu^2+^ groups, respectively. After that, the concentrations of Chla gradually increased and at the end of the experiment (144 h) they increased to 2240 ± 123, 2198 ± 46, and 1652 ± 68 μg/L for the control, 0.5 mg/L and 2 mg/L Cu^2+^ groups, respectively. A delay response of quantum yield in periphyton was observed within the first 24 h, and then a significant decrease of quantum yield was observed in the 2 mg/L Cu^2+^ treated periphyton (Figure 3B).

The dramatic decreases in the total Chla amounts and quantum yield in Cu^2+^ exposure groups were due to the toxicity of Cu^2+^ on algae, which caused the death of algae [23,24]. In addition, the algae in periphyton will have the adaption time at the beginning, so the decrease was also observed in the control, which was also observed in the previous study [25]. After the adaption of periphyton, the algae will regenerate with the reduction of Cu^2+^ and its toxic effects, therefore the photoautotrophs can resume and the total Chla concentration can increase [19,23]. However, after 144 h incubation, the concentration of total Chla in the 2 mg/L Cu^2+^ treatment was obviously lower than the control and 0.5 mg/L Cu^2+^, indicating the obvious inhibition of 2 mg/L Cu^2+^ on periphyton biomass. Previous studies have indicated that Cu^2+^ is a fundamental element in microbial enzymes, involving respiration, connective tissue biosynthesis and other bioprocess [17]. The excessive Cu^2+^ exposure may lead to the metal toxicity by destroying cell integrity, decreasing enzymes activity and so on [23]. Due to the complex community structure, periphyton gradually adjusted microbial community structure to respond to Cu^2+^ stress and exhibited consecutive Cu^2+^ removal.

### 3.3. Effect of Cu^2+^ on Carbon Metabolism of Periphyton

**Figure 4:**
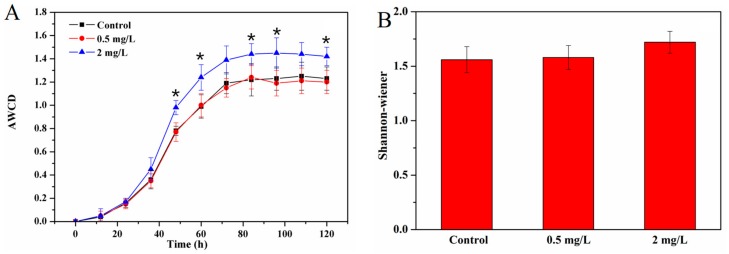
The average well color development (AWCD) of all carbon sources in periphyton communities treated with different Cu concentrations within incubation time (**A**) and Shannon-Wiener (metabolic diversity indices) for periphyton (**B**). * indicates significant differences at *p* < 0.05 between the control and Cu^2+^ treatments.

After the Cu treatments, the changes of carbon metabolism of periphyton, represented by AWCD, were determined using Biolog Eco microplates. Accordingly, AWCD can be used to analyze the carbon metabolic activity of heterotrophic microorganisms, which revealed the features of their metabolic functions since different species are able to utilize different carbon sources [29,31]. The higher value of AWCD means stronger microbial activity and abilities for organic matter utilization. In this study, an obvious lag period of the AWCD was observed for all the treatments within the first 24 h (Figure 4). Then, the AWCD in periphyton started to increase significantly with time, suggesting that the periphyton exhibit the ability to metabolize organic matters in Biolog Eco microplates. The AWCD reached the peak after 84 h and then the metabolic utilization capability of periphyton tended to be steady. Similar research and methods were reported in previous studies [25,30]. 

After the Cu^2+^ treatments, 0.5 mg/L Cu^2+^ had no obvious effects on the metabolic rate and the AWCD in periphyton (*p* > 0.05), suggesting that low concentration exposure of Cu^2+^ does not affect the carbon source utilization of periphyton. The metabolic rate in 2 mg/L Cu^2+^ treatment was higher than the other two treatments. The AWCD of 2 mg/L Cu^2+^-treated periphyton increased from 0 to around 1.45 after 84 h, higher than those in the control and 0.5 mg/L Cu^2+^-treated periphyton groups (*p* < 0.05). These results indicated that 2 mg/L Cu^2+^ stimulated the carbon source utilization of periphyton. Previous studies have reported that the 2 mg/L Cu^2+^ exposure increased the carbon metabolic activity of freshwater biofilms, while a strong inhibition effect was observed with Cu^2+^ concentration increased to 10 mg/L [25].

In addition, the index of Shannon-Wiener was used to compare the metabolic diversity in periphyton, which was calculated based on the profiles at 84 h (Figure 4). As shown in Figure 3B, the Shannon-Wiener was 1.56 ± 0.12, 1.58 ± 0.11 and 1.72 ± 0.1 for the control, 0.5 mg/L and 2 mg/L Cu^2+^ treatments, respectively. Compared with the control, Cu^2+^ exposure had no significant effect on metabolic diversity in periphyton, even after 2 mg/L Cu^2+^ exposure. Moreover, 2 mg/L Cu^2+^ slightly increased the value of Shannon-Wiener (*p* > 0.05), suggesting that Cu^2+^ exposure might increase metabolic processes in periphyton. This was consistent with the results observed in the AWCD measurements. Yang et al. [5] reported a similar conclusion that Cu^2+^ could significantly change the ability of bacteria to utilize carbon sources. Our study illustrated that metabolic activity of microbial community and utilization of carbon sources in periphyton could keep at normal levels or even increase after exposure to Cu^2+^. The results indicated that periphyton had the high resistance on Cu^2+^ and could sustain their microbial activity [23,32], suggesting that periphyton might be an environmentally friendly medium to remove high Cu^2+^-polluted water.

### 3.4. Pollutants Removal of Periphyton after Exposed to Cu^2+^

**Figure 5:**
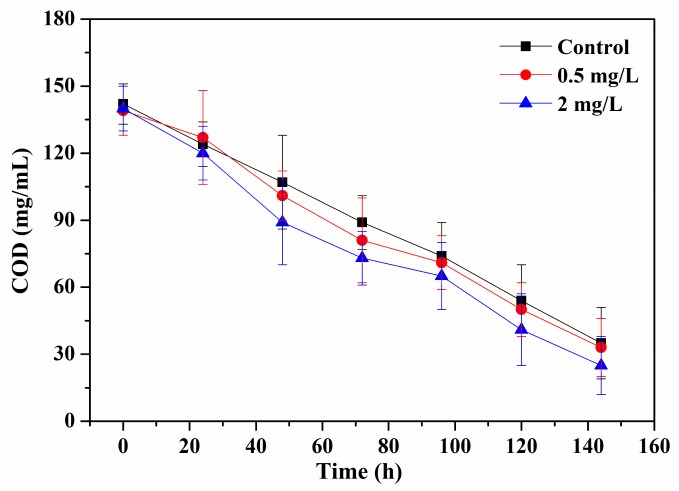
Dynamic changes of chemical oxygen demand in synthetic wastewater by periphyton after exposure to 0.5 and 2 mg/L Cu^2+^, and the control.

The above results showed that periphyton could remove Cu^2+^ from water effectively and exhibited higher resistance on Cu^2+^ exposure. To evaluate the function of periphyton after exposure to Cu^2+^, periphyton was cultured in artificial wastewater and COD were measured to monitor their ability in degrading organic matter. Figure 5 shows that periphyton treated with 0.5 mg/L Cu^2+^ had a similar COD removal rate with the control group, and 2 mg/L Cu^2+^ slightly decreased the COD removal rate without statistical difference. Specifically, the concentrations of COD were reduced to 35 ± 16, 33 ± 13 and 25 ± 13 mg/L after 144 h for the control, 0.5 mg/L, and 2 mg/L Cu^2+^, respectively. These results suggested the ability of degradation of organic matter in periphyton was not affected and periphyton could still keep their normal function after exposure to Cu^2+^.

Studies have showed that periphyton could maintain or regain microbial function through resistance and functional redundancy [20,33,34]. In this study, after exposure to Cu^2+^, the algae community structure in periphyton was changed, however, the metabolic activity and the COD removal rate of periphyton was kept even after exposure to 2 mg/L Cu^2+^. These results suggested that periphyton could exhibit strong resistance to heavy metals at various concentrations [23] and periphyton can serve as a promising regulator of heavy metal removal in water environments [9]. 

## 4. Conclusions

In this study, high Cu removal efficiencies were observed by periphyton with various concentrations (0.5 to 2 mg/L). Two milligrams per liter of Cu^2+^ obviously decreased the amounts of total chlorophyll *a* indicating that 2 mg/L Cu^2+^ inhibited microalgal growth. Meanwhile, the metabolic activity (heterotrophic microbes) and the COD removal rate of periphyton was maintained after being treated with 2 mg/L Cu^2+^. Overall, the results showed that periphyton exhibits strong tolerance on Cu stress, which can remove Cu^2+^ from wastewater and sustain their microbial functions at the same time.

## Supplementary Materials

The following are available online at https://www.mdpi.com/1660-4601/17/3/941/s1, Test S1: Determination of Chla and quantum yield of periphyton; Test S2: Determination of microbial metabolic activity of periphyton.
