# Supplementary material for: Responses of Periphyton Microbial Growth, Activity, and Pollutant Removal Efficiency to Cu Exposure
Source: Int J Environ Res Public Health. 2020 Feb 3;17(3):941. doi: 10.3390/ijerph17030941 (PMC7037227; doi:10.3390/ijerph17030941)
Supplement: Supplementary file 1 [file ijerph-17-00941-s001.pdf]

International Journal of
Environmental Research and Public Health

MDPI

Article

Responses of Periphyton Microbial Growth, Activity, and Pollutant Removal Efficiency to Cu Exposure

Wei Zhong, Weiqun Zhao and Jianhui Song

Supplementary Material

Test S1 Determination of Chla and quantum yield of periphyton

The periphyton samples were scraped off using a sterile brush. Then, 0.5 g of wet periphytic biofilm was suspended in 60 mL 0.85% sterile NaCl solution and shaken for 30 min at 150 rpm. The Chla concentration and quantum yield of periphyton suspension were measured using the PHYTO-PAM-II according to the procedures described in previous study [27,28]. A 15-min dark adaption was conducted prior to the determination. The fluorescence attributed to cyanobacteria, green alga, diatom and cryptophyta referred to Fo (Bl), Fo (Gr), Fo (Br) and Fo (PE-Type), respectively, which can represent the relative contribution of each microalgal group to the total community.

Test S2 Determination of microbial metabolic activity of periphyton

The microbial metabolic activity of periphytic biofilms were measured using BiologTM ECO Microplates at 0, 12, 24, 36, 48, 60, 72, 84, 96, 108, and 120 h during the incubation [29,30]. 0.1 g of wet biofilm sample was suspended in 50 ml 0.85% sterile NaCl solution and shaken for 30 min at 150 rpm. After a 10-min settling, 150 μL aliquot was pipetted into each well of the ECO microplate and then incubated at 20 °C. The absorbance was measured with Synergy H4 Microplate Reader at every 12 h for 120 h at 590 nm wavelength. All measurements were done in three replicates as the 96 well system consists of three times the 31 carbon sources and the control well. Values lower than the control well were set to same value as control.
